# Analysing biomarkers in oral fluid from pigs: influence of collection strategy and age of the pig

**DOI:** 10.1186/s40813-023-00333-x

**Published:** 2023-08-30

**Authors:** Mario Andre S. Ornelas, María José López‑Martínez, Lorena Franco-Martínez, José J. Cerón, Alba Ortín-Bustillo, Camila Peres Rubio, Edgar Garcia Manzanilla

**Affiliations:** 1 Pig Development Department, Teagasc Grassland Research and Innovation Centre, Moorepark, Fermoy, Co. Cork Ireland; 2 School of Veterinary Medicine, University College Dublin, Belfield, Dublin, Ireland; 3 Interdisciplinary Laboratory of Clinical Analysis, Regional Campus of International Excellence ‘Campus Mare Nostrum’, University of Murcia (Interlab-UMU), University of Murcia, Campus de Espinardo s/n, Murcia, 30100 Spain

**Keywords:** Analyte, Health and welfare, Saliva, Sample collection, Swine

## Abstract

**Background and objectives:**

Oral fluid (OF) is an easy-to-collect, inexpensive, fast and non-invasive sample to characterize health and welfare status of the pig. However, further standardisation of the collection methods is needed in order to use it regularly in veterinary practice. Cotton ropes are routinely used to collect OF for pathogen detection but they may not be optimal for biomarker analysis due to sample contamination. This study compared two methods (cotton ropes and sponges) to collect porcine OF for biomarker analysis. A panel of 11 biomarkers of stress, inflammation, sepsis, immunity, redox status and general homeostasis was studied.

**Materials and methods:**

Eighteen farrow-to-finish pig farms were included in the study. In each farm, three (for sponges) or four pens of pigs (for ropes) were sampled at four age categories: the week after weaning (5 weeks), before (11–12 weeks) and after (12–13 weeks) moving to finisher facility and the week before slaughter (22–25 weeks). In total, 288 OF samples were collected with cotton ropes and 216 with sponges and analysed for the biomarkers: cortisol, alpha-amylase, oxytocin (stress), haptoglobin (inflammation), procalcitonin (sepsis), adenosine deaminase, immunoglobulin G (immune system), ferric reducing antioxidant power (redox status), and creatine kinase, lactate dehydrogenase and total protein (general homeostasis). Samples were also scored visually for dirtiness using a score from 1 (clean) to 5 (very dirty).

**Results:**

Rope-collected OF had higher levels of dirtiness (3.7 ± 0.04) compared to sponge-collected OF (2.7 ± 0.15) and had higher values than sponges for cortisol, procalcitonin, oxytocin, haptoglobin, total protein, lactate dehydrogenase and ferric reducing antioxidant power. All biomarkers decreased in value with age. Immunoglobulin G did not perform well for any of the two collection methods.

**Discussion and conclusion:**

The results showed a clear effect of age on the biomarkers in OF collected with both, sponges or ropes. Sponges provided a cleaner sample than cotton ropes for biomarker analysis. Both methods are easy to apply under the commercial conditions in pig farms although sponges may take more time in early weaner stages. From a practical point of view, sampling with sponges achieved the best combination of reduced sampling time and low contamination.

## Background

Diagnostic tools that are easy to use under commercial farming conditions and that allow for a quick assessment of the health and welfare status of a group of pigs can be valuable instruments for farmers and veterinarians. When selecting these tools, in addition to the intrinsic characteristics of the tool like sensitivity and specificity, it is important to consider how well these tools are suited for field conditions. Factors to consider include cost, training requirements, labour, evaluation time and potential health and welfare implications for the animal in the case of invasive methods [1–3]. Oral fluid (OF) has a wide range of applications as a diagnostic sample and is cheap and easy to collect without compromising animal welfare [4, 5]. Sample collection is particularly simple in pigs because they voluntarily chew on sample-collecting devices [4, 5]. The lack of training required to collect OF is another important advantage as any farm staff can do it, contrary to blood collection, which normally requires a veterinarian. Although information on OF collection is not abundant, it usually involves using low-cost absorptive devices such as cotton ropes [6] or sponges [7].

Other than saliva, OF contains transudates from the circulatory system and oro-naso-pharyngeal secretions [8–10]. Thus, pathogens, antibodies and biomarkers of interest may be present in this biological sample, making it useful for disease surveillance and diagnostic purposes [6, 8, 10]. Numerous biomarkers in porcine OF have been validated for indicators of stress, like chromogranin A or D-dimer [11, 12], inflammation, like acute phase proteins [10], immunity, like adenosine deaminase [13] or redox status, like ferric reducing capacity of plasma [14]. In order to use this information to measure the health and welfare status of a pig herd, it is crucial to standardize methods for sample collection, processing and analysis. The methods for analysis and the applications of OF keep expanding, however, the methods to collect and process the samples usually receive little attention. Olsen et al. (2013) reported that both, type of collection device and sample processing (centrifuging and filtration) influence OF testing results [15]. As the detrimental effect of sample contamination on test performance has been highlighted in other studies [16–18] this study compared two methods to collect OF for biomarker measurements in pigs at different ages and discussed practical considerations of OF sampling under the commercial farming conditions.

## Methods

All procedures carried out in this study were approved by the Teagasc Animal Ethics Committee (reference 0123–368). Teagasc is the Irish Agriculture and Food Development Authority and carries out research, knowledge transfer and education at a national level in the Republic of Ireland.

### Farms and sampling

Eighteen farrow-to-finish Irish pig farms (farm size range 130 to 2400 sows) were visited as part of a cross-sectional study. Each farm was visited once to sample pigs for OF in the following ages: one week after weaning (W1), one week prior to transfer to the finishing facility (W2), one week after transfer to the finishing facility (F1) and one week before slaughter (F2). Weaning in Irish pig farms normally takes place between 28 and 32 days of age, pigs are moved to finishing facility around 12 weeks of age and are sent to slaughter between 22 and 25 weeks of age when they reach 110 to 115 kg of live weight.

Sampling was carried out at a pen level using 2 devices, ropes and sponges. The number of samples needed was calculated taking into account the variability of each method in preliminary data. Four ropes from four different pens and three sponges from three different pens were collected for each age. In total, 16 samples were collected with ropes and 12 samples were collected with sponges per farm.

### Oral fluid collection and processing

**Fig. 1:**
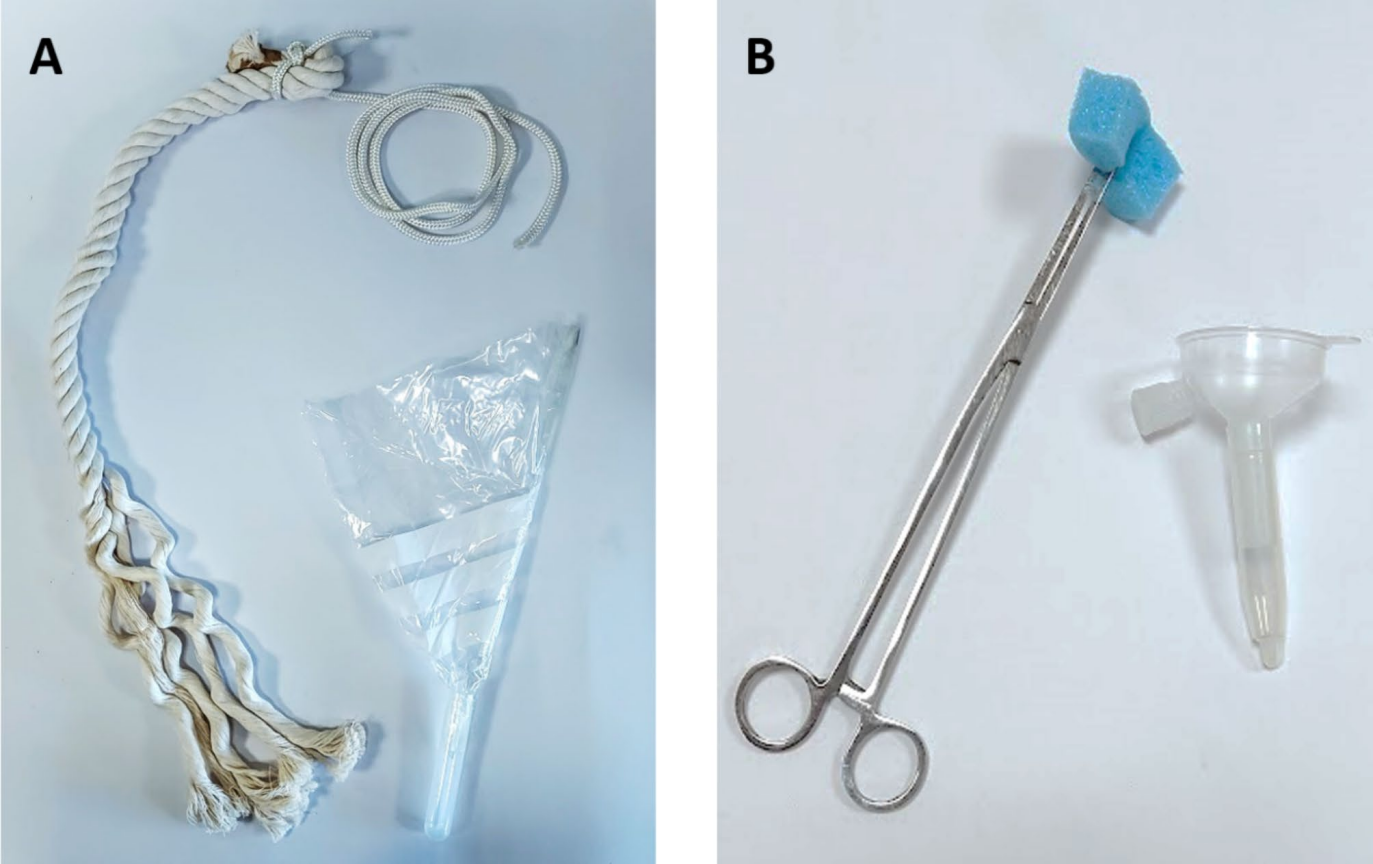
Devices used for oral fluid collection and extraction into tubes. Cotton ropes were hung at shoulder height and later squeezed to a tube container (**A**); sponges were held with a forceps and later transferred to a Salivette tube (Sarstedt®, Nümbrecht, Germany) with a funnel (**B**)

Two methods to collect OF were used. The first method consisted of hanging a cotton rope (Hipra, Amer, Spain) at shoulder height. In order to minimise sample c ontamination, ropes were hung away from pen walls, feeder and drinker, or held by an operator. Oral fluid was extracted from ropes by transferring these into a plastic bag attached to a 10 mL tube in one open corner and squeezing them (Fig. 1a).

The second collection method used a sponge (45 mm x 25 mm x 25 mm; Esponja Marina, La Griega E. Koronis, Madrid, Spain) held with a forceps and exposed to the pen of pigs. After sample collection, the sponge was transferred to a Salivette tube (Sarstedt®, Nümbrecht, Germany) with the help of a funnel (Fig. 1b). Both collection devices were offered to be chewed on until significantly moist and the extracted OF samples were refrigerated. All sampling material were replaced or cleaned after each collection to avoid cross-contamination. Samples were then centrifuged at 3000* g* for 5 min and the supernatant frozen at -20 °C until further analysis.

### Biomarker measurements and sample dirtiness

Each sample was assessed for contamination using a qualitative scale from 1 to 5 based on the visual inspection of the sample colour, as described in Franco-Martínez et al. (2022) [17]. The biomarkers analysed for all samples were cortisol, alpha-amylase and oxytocin as indicators of stress, haptoglobin as an indicator of inflammation, procalcitonin as an indicator of sepsis, adenosine deaminase (ADA) and immunoglobulin G (IgG) as indicators of immune activation, ferric reducing antioxidant power (FRAP) as an indicator of redox status, and creatine kinase (CK), lactate dehydrogenase (LDH) and total protein as indicators of general homeostasis. These biomarkers were measured using the methods described in Table 1.

**Table 1:** List of methods used to analyse the biomarkers included in the trial

Biomarker	Method	Reference
Stress		
Cortisol	In house AlphaLISA assay using a commercial monoclonal antibody	Lopez-Arjona et al., 2020 [19]
Amylase	Commercial spectrophotometric assay	Fuentes et al., 2011 [20]
Oxytocin	In house AlphaLISA assay using an in house monoclonal antibody	Lopez-Arjona et al., 2020 [19]
Inflammation		
Haptoglobin	In house AlphaLISA assay using an in house monoclonal antibody	Contreras-Aguilar et al., 2021 [21]
Sepsis		
Procalcitonin	In house AlphaLISA assay using an in house polyclonal antibody	López-Martínez et al. 2022 [22]
Immune system		
ADA	In house spectrophotometric assay	Tecles et al. 2018 [13]
Immunoglobulin G	Commercial sandwich Elisa	Navarro et al. 2021 [23]
Oxidative stress		
FRAP	In house spectrophotometric assay	Rubio et al. 2019 [14]
General homeostasis		
Protein	Commercial colorimetric kit	Ortín-Bustillo et al. 2022 [7]
Creatine Kinase	Commercial colorimetric kit	Ortín-Bustillo et al. 2022 [7]
LDH	Commercial colorimetric kit	Ortín-Bustillo et al. 2022 [7]

Note: ADA: Adenosine deaminase; LDH: Lactate dehydrogenase; FRAP: Ferric reducing antioxidant power

### Statistical analysis

All data were processed and analysed using R version 3.5.1, including R packages lme4 version 1.1.21 and ggplot2 version 3.2.1. The distribution of each biomarker measurement was checked for normality using graphical methods and the Shapiro-Wilk test. A logarithmic transformation was used for all non-normally distributed data. For each biomarker and for the contamination score, a general linear regression model was adjusted including fixed factors age, collection device and the interaction between the two variables. Farm was considered as a random factor for a model when significant. Correlation analysis was also used to study the relationship between both collection devices. Results are expressed as mean ± standard error and alpha level for determination of significance was 0.05.

## Results

A total of 503 OF samples were analysed, of which 288 were collected with ropes and 215 with sponges. Sample colour (1–5), as an indicator of contamination, was higher in rope-collected OF (3.7 ± 0.04) in comparison with sponge-collected samples (2.7 ± 0.15) (Table 2). Ropes collected in W1 were cleaner than those collected at later ages for each device. Biomarkers were analysed in all samples, except for IgG and CK measurements. Only 7% and 56% of rope-collected and sponge-collected samples, respectively, were successfully analysed for IgG and 83% of rope-collected samples for CK.

**Table 2:** Relative frequencies and average values of sample color (1–5) as a qualitative indicator of sample contamination

	W1 5 weeks old		W2 11 weeks old		F1 13 weeks old		F2 24 weeks old	
Device Colour	Sponge (n = 54)	Rope (n = 72)	Sponge (n = 53)	Rope (n = 72)	Sponge (n = 54)	Rope (n = 72)	Sponge (n = 54)	Rope (n = 72)
**1 (1.8%)**	9.3%		1.9%				5.6%	
**2 (21%)**	57.4%		43.4%	4.2%	40.7%		48.1%	
**3 (40.2%)**	27.8%	45.8%	41.5%	30.5%	53.7%	37.5%	46.3%	40.3%
**4 (31%)**	5.5%	54.2%	7.5%	37.5%	5.6%	55.6%		56.9%
**5 (6%)**			5.7%	27.8%		6.9%		2.8%
Mean score ± SEM	2.3^d^ ± 0.1	3.5^b^ ± 0.1	2.7^c^ ± 0.1	3.9^a^ ± 0.1	2.7^c^ ± 0.1	3.7^ab^ ± 0.1	2.4^ cd^ ± 0.1	3.6^ab^ ± 0.1

Cell colour (ranging from dark green to red) is to be interpreted as a heat map of the concentration of samples in each category. The percentage values indicate the proportion of samples in each category per age. The colors take into account all 503 samples collected with both devices. W1: one week after weaning; W2: one week before being transferred to finisher facility; F1: one week after being transferred to the finishing facility; F2: one week before slaughter.

The results for the 11 analysed biomarkers according to age and sample collection device are reported in Table 3. All biomarkers showed differences in their mean values by age and collection device except for immunoglobulin G that showed no differences between devices. All biomarker values decreased with age (P < 0.001). Samples collected with ropes showed higher values than sponges for cortisol (P < 0.001) procalcitonin (P < 0.001), oxytocin (P < 0.001), haptoglobin (P < 0.001), total protein (P < 0.001), LDH (P < 0.001), and FRAP (P < 0.001). The opposite was observed for ADA (P < 0.001), amylase (P < 0.002) and CK (P < 0.001). For cortisol, haptoglobin and ADA, an interaction between age and device was present. In the case of cortisol the drop with ages was different between ropes and sponges. For haptoglobin, rope values were only higher in F2. For ADA, sponge values were higher in W1 and W2.

**Table 3:** Results of 11 biomarkers measured in oral fluid samples collected from 18 farms at four different ages

Biomarker	W1	W2	F1	F2	Average	X	P-value
Cortisol, ng/mL Sponge (N = 215) Rope (N = 286) Average	75^ cd^ ± 7 161^a^ ± 10 118^a^	48^de^ ± 6 124^ab^ ± 9 86^b^	31^f^ ± 4 84^bc^ ± 5 57^c^	40^ef^ ± 7 80^c^ ± 7 60^c^	48^b^ 112^a^	Age Device Age*Device	< 0.001 < 0.001 0.006
Procalcitonin, µg/mL Sponge (N = 214) Rope (N = 288) Average	6.3 ± 0.7 24.6 ± 2.1 15.4^a^	4.3 ± 0.9 16.2 ± 1.7 10.2^b^	2.8 ± 0.3 8.6 ± 0.6 5.7^c^	2.8 ± 0.3 7.1 ± 0.6 4.9^c^	4.1^b^ 14.1^a^	Age Device Age*Device	< 0.001 < 0.001 0.056
Oxytocin, ng/mL Sponge (N = 213) Rope (N = 287) Average	2.3^c^ ± 0.3 4.9^a^ ± 0.5 3.6^a^	2.2^ cd^ ± 0.3 3.9^ab^ ± 0.4 3.1^ab^	1.7^de^ ± 0.3 2.8^bc^ ± 0.2 2.3^bc^	1.2^e^ ± 0.1 2.4^c^ ± 0.2 1.8^c^	1.8^b^ 3.5^a^	Age Device Age*Device	< 0.001 < 0.001 0.947
Haptoglobin, µg/mL Sponge (N = 214) Rope (N = 288) Average	5.2^a^ ± 0.4 4.4^a^ ± 0.2 4.8^a^	3.5^b^ ± 0.4 3.6^ab^ ± 0.2 3.5^b^	3.2^b^ ± 0.3 3.7^ab^ ± 0.2 3.4^b^	1.4^d^ ± 0.2 1.9^c^ ± 0.1 1.6^c^	3.3^b^ 3.4^a^	Age Device Age*Device	< 0.001 < 0.001 0.004
ADA, IU/mL Sponge (N = 215) Rope (N = 288) Average	3.8^a^ ± 0.27 1.9^b^ ± 0.56 2.9^a^	1.7^b^ ± 0.11 1.2^ cd^ ± 0.04 1.5^b^	1.7^bc^ ± 0.11 1.2^ cd^ ± 0.03 1.4^b^	1.2^d^ ± 0.09 1.1^d^ ± 0.04 1.1^c^	2.1^a^ 1.3^b^	Age Device Age*Device	< 0.001 < 0.001 < 0.001
Amylase, IU/mL Sponge (N = 215) Rope (N = 288) Average	1.8 ± 0.29 1.2 ± 0.11 1.5^a^	0.9 ± 0.16 0.8 ± 0.06 0.8^b^	1.0 ± 0.24 0.6 ± 0.04 0.8^b^	0.5 ± 0.16 0.5 ± 0.04 0.5^c^	1.0^a^ 0.8^b^	Age Device Age*Device	< 0.001 0.002 0.135
Total protein, mg/dL Sponge (N = 213) Rope (N = 287) Average	232 ± 21 376 ± 25 304^a^	136 ± 10 233 ± 11 185^b^	118 ± 7 224 ± 8 171^b^	95 ± 5 178 ± 8 137^c^	145^b^ 253^a^	Age Device Age*Device	< 0.001 < 0.001 0.709
IgG, µg/mL Sponge (N = 120) Rope (N = 21) Average	1.1 ± 0.22 0.8 ± 0.29 0.9^a^	1.2 ± 0.26 1.7 ± 0.72 1.5^a^	1.1 ± 0.25 0.5 ± 0.43 0.8^ab^	0.4 ± 0.08 0.7 ± 0.67 0.5^b^	0.9 0.9	Age Device Age*Device	< 0.001 < 0.398 0.530
LDH, IU/mL Sponge (N = 213) Rope (N = 275) Average	0.6 ± 0.07 1.5 ± 0.12 1.0^a^	0.2 ± 0.04 0.4 ± 0.03 0.3^b^	0.2 ± 0.02 0.5 ± 0.06 0.3^b^	0.1 ± 0.01 0.3 ± 0.05 0.2^c^	0.3^b^ 0.7^a^	Age Device Age*Device	< 0.001 < 0.001 0.430
FRAP, µmol/L Sponge (N = 213) Rope (N = 288) Average	690 ± 57 2076 ± 110 1383^a^	651 ± 83 1770 ± 137 1210^b^	551 ± 51 1536 ± 81 1044^b^	419 ± 31 1374 ± 84 897^b^	578^b^ 1689^a^	Age Device Age*Device	< 0.001 < 0.001 0.895
Creatine Kinase, IU/L Sponge (N = 210) Rope (N = 239) Average	20.0 ± 1.7 16.0 ± 1.9 17.9^a^	9.4 ± 0.1 8.8 ± 1.1 9.1^b^	9.7 ± 0.8 10.7 ± 0.8 10.2^b^	5.2 ± 0.5 5.3 ± 6.7 5.2^c^	11.0^a^ 10.2^b^	Age Device Age*Device	< 0.001 0.003 0.064

Age and device effects are shown in the average row and column. means with. No common letter in the superindex for age (row) and device (column) are different. When interaction was significant, letters are also provided for the non-average figures and any two means (row or column) with no common superindex are different. ADA: Adenosine deaminase; IgG: Immunoglobulin G; LDH: Lactate dehydrogenase; FRAP: Ferric reducing antioxidant power; W1: one week after weaning; W2: one week before being transferred to finisher facility; F1: one week after being transferred to the finishing facility; F2: one week before slaughter

The correlation between biomarker measurements for the two collection devices across the different ages is reported in Table 4. With the exception of haptoglobin at W1 (r = 0.53), results did not show good correlations (r < 0.50) between sample collection devices. Due to the reduced number of measurements of IgG in rope-collected samples, this analyte was not included in this part of the analysis.

**Table 4:** Correlation between biomarker values for samples collected using sponges and ropes analysed by age and pooled for all ages

Biomarker	W1	W2	F1	F2	All ages
Cortisol	0.32	− 0.21	0.12	− 0.01	0.00
Procalcitonin	− 0.18	0.42	-0.07	− 0.17	− 0.06
Oxytocin	− 0.35	− 0.10	− 0.17	− 0.11	− 0.04
Haptoglobin	0.53*	0.21	− 0.16	0.22	0.10*
ADA	-0.07	0.17	0.00	− 0.03	− 0.08
Amylase	0.16*	0.15	− 0.28	− 0.13	− 0.09
Total protein	− 0.17	0.26*	− 0.02	− 0.13*	− 0.06
LDH	0.27	0.43*	0.28	0. 26	0.15*
FRAP	0.14	0.30*	− 0.06	0.09	0.06
Creatine Kinase	0.26*	-0.12	0.17	0.22	-0.06

ADA: Adenosine deaminase; LDH: Lactate dehydrogenase; FRAP: Ferric reducing antioxidant power; W1: one week after weaning; W2: one week before being transferred to finisher facility; F1: one week after being transferred to the finishing facility; F2: one week before slaughter; an asterisk indicates a significant result

## Discussion

Using OF in pigs is very convenient because it is a cheap, non-invasive and easy to collect sample thanks to the natural curious behaviour of pigs. Thus, the development of biomarker analysis in OF is a very promising option to assess the health and welfare of pig populations. However, methods need to be further tested and standardised. This study compared two methods, cotton ropes and sponges, to collect OF samples in pig farms to measure biomarkers at different ages. We hypothesised that, although cotton ropes are regularly used to collect OF for pathogen detection [6] in veterinary practice, sponges may be a better alternative in practice because of the high contamination of ropes.

Franco-Martínez et al. (2022) showed, in lab conditions, that the performance of biomarker analyses in OF is affected by the contamination of the sample [17], in particular by faeces and feed. This is relevant for OF samples collected with ropes because this device tends to retain a significant amount of dust and dirt from the environment. A panel of 11 biomarkers of stress, inflammation, sepsis, immunity, redox status and general homeostasis previously used by Franco-Martínez et al. was studied in the current study for samples collected with cotton ropes and sponges in 18 commercial pig farms. There was a marked difference in the values of most of the biomarkers between collection devices, most likely due to contamination of the device. Extraction of the OF into tube containers was similar between devices and this is an improbable source of bias because sample manipulation was minimal. Post-collection sample processing can affect the measurement of analytes, such as immunoglobulins, as reported by Olsen et al. (2013) [15]. However, the methods to centrifuge, store and analyse all samples in the current study were uniform. Sample colour (1–5), a qualitative indicator of contamination [17], was higher in rope-collected samples compared to sponge-collected samples (means: 3.7 vs. 2.7), regardless of the age of the pigs. This increase in colour is probably a good indicator of contamination in this case; however, a lack of change in colour would not necessarily mean an absence of contamination. Previous reports found that the type of rope material (cotton vs. hemp vs. nylon) can be associated with the performance of the analyses [15]. Differences between cotton ropes and sponges for collection of OF for biomarker analysis were not described yet but the current study shows very clear effects of the sampling method.

Results differed also across ages, with biomarker values decreasing along the production cycle for both collection devices as previously reported by Bustillo et al. (2022) using sponges in an experimental farm [7]. The selection of sampling ages for this trial was done because W1 and F1 are periods of stress for the pigs. In this periods, pigs move from lactation to weaner facilities and from weaner to finisher facilities, respectively. The movement from lactation to the weaner facilities is the most stressing moment of the life of a pig as shown by the highest values in biomarkers. This study also included an additional sampling time point during lactation to study the changes in biomarkers values due to weaning. However, this sampling was abandoned because of the lack of interest of the animals for the devices in the lactation facility. Sampling at this age required manipulation of the animals and often induced contamination of the sample with blood and was abandoned after the second farm sampling.

Previous studies generally used animals from a single age group, mainly finishers [24, 25]. Bustillo et al. (2022) characterized a panel of 29 OF biomarkers from 49 pigs from a single origin, throughout five ages (lactation, followed by the 4 ages in the present report). In addition to a decrease on values with age, as in the current study, Bustillo et al. also grouped biomarkers by pattern of result distribution across time. All 9 biomarkers used in the two studies had higher concentrations after weaning compared with the later three ages in both studies. These are cortisol, oxytocin, haptoglobin, ADA, amylase, protein, LDH, FRAP and CK.

Unlike most studies, carried out on a single experimental farm and focused on a limited number of animals, the current work focused on larger animal populations from 18 different farms and with a very practical approach. This should be taken into account when comparing the results obtained here with values from other reports.

From a practical point of view, sampling with sponges achieves the best combination of reduced sampling time and low contamination. This is applicable to all ages except for weaners W1, where obtaining a single sample from a sponge may require holding the device for up to 15 min. However, sampling W1 with ropes takes also 15 to 30 min per pen. In the later three ages (W2, F1, F2), sponges rarely took more than one minute to obtain at least 1ml of sample. In terms of sampling with cotton ropes, an exposure time of 5 min allowed the collection of at least 10mL of sample from W2, F1, F2. Ropes do not require the constant presence of an operator but often the veterinarian needs to access the pen to place them in optimal position, contrary to sponges.

Regarding sampling figures, both sampling methods were carried out at pen level and not targeting a fixed number of pigs. It is difficult to know how many pigs interacted with each method. For weaners, pens were generally bigger (14 to 100 piglets per pen) and the number of piglets that interacted with a rope within a pen was normally higher than the number of piglets that interact with the sponges. Five to ten piglets typically chewed a sponge and more than 15 piglets chewed ropes in big pens. In the case of finishers, numbers were more similar between methods because the number of pigs per pen were lower, pigs were bigger and usually 5–10 pigs interacted with both devices. This difference may affect the results of the comparison but in the current trial we decided to use the methods as they would be used in practice to account for the intrinsic limitations of the methods.

## Conclusions

The biomarkers of stress, inflammation, sepsis, immunity, redox status and general homeostasis studied in porcine oral fluid displayed variability in measurements according to collection device and age. These variables should be considered when interpreting biomarker results. From a practical point of view, sampling with sponges achieved the best combination of reduced sampling time and low contamination. Further investigations are needed to understand how the porcine biomarker profile in oral fluid is affected by health, welfare and other production related variables.

## Data Availability

The datasets used in this study are available from the corresponding author upon reasonable request.

## Abbreviations

ADA: Adenosine deaminase
CK: Creatine kinase
F1: One week after transfer to the finishing facility
F2: One week before slaughter
FRAP: Ferric reducing antioxidant power
Hapt: Haptoglobin
IgG: Immunoglobulin G
LDH: Lactate dehydrogenase
OF: Oral fluid
PCT: Procalcitonin
Pr_Or: Protein
W1: One week after weaning
W2: One week prior to transfer to the finishing facility
